# Re: Are you Discussing Dental Caries in Children with Current and Local References? - Letter to The Editor

**DOI:** 10.31729/jnma.3708

**Published:** 2018-10-31

**Authors:** Parajeeta Dikshit

**Affiliations:** 1 Department of Pedodontics, Kantipur Dental College Teaching Hosspital and Research Centre, Kathmandu, Nepal

**Dear Editor**,

I would like to thank the reader for taking a keen interest in our article titled “Relationship of Body Mass Index with Dental Caries among Children attending Pediatric Dental Department in an Institute”.^1^ I acknowledge their valuable remarks and suggestions positively and thank them immensely for taking interest in going through our article.

The research aimed only to determine the relationship of nutrition status with dental caries among children and not discuss Dental caries in children with current and local references.

My response to their constructive comments are as follows:

1. The authors had asked “why the authors have highlighted in the discussion section that “… the caries prevalence in children has not shown a significant decline”. This statement is not correct.” Globally there is stated to be decline in dental caries but it is not significant as the decline is seen in developed countries while the developing countries show a varying trend. Mehta A^2^ have shown in their retrospective study using data of 25 years that the trend varied with increase in certain age groups. The National Pathfinder survey of Nepal 20 04^3^ shows that 57.5% of five to six years age group and 25.6% of 12 to 16 years age group suffer from dental caries. While the prevalence of dental caries in a study conducted in Nepal in 2017 by Khanal et al^4^ shows a prevalence of 62 % and Khanal etl al 2014^5^ found 58.3 % prevalence of dental caries among 12 to 15 years old children. The article suggested by the authors:comprehensive review conducted by Frencken et al^6^ “states the limitation of incompleteness of the data in the WHO databank, with few studies included for the low-income countries' group. They have further stated that the variations exist in prevalence and severity between high and low-income countries and in the prevalence of open cavitated dentine carious lesions. Dental caries being less prevalent in high-income than in low and lower-middle-income countries among adolescents and adults”.
2. The authors further pointed out that the prevalence of dental caries mentioned in our article different from the researches suggested by them. The research that they have suggested in the table are community based while the present one was done in the dental hospital where parents visit after they encounter a problem and this reason has already been explained in our article itself. Similar prevalence has been reported by Upadhyay et al.^7^ in a study conducted in dental college where 91.3% children had dental caries. World Health Organization reports 60–90% of school children worldwide have experienced caries, with the disease being most prevalent in Asian and Latin American countries.

## Conflict of Interest

**None.**
